# Genetic predisposition toward suicidal ideation in patients with acute coronary syndrome

**DOI:** 10.18632/oncotarget.21661

**Published:** 2017-10-07

**Authors:** Hee-Ju Kang, Kyung-Yeol Bae, Sung-Wan Kim, Il-Seon Shin, Young Joon Hong, Youngkeun Ahn, Myung Ho Jeong, Jin-Sang Yoon, Jae-Min Kim

**Affiliations:** ^1^ Department of Psychiatry, Chonnam National University Medical School, Gwangju, Korea; ^2^ Department of Cardiology, Chonnam National University Medical School, Gwangju, Korea

**Keywords:** acute coronary syndrome, suicidal ideation, polymorphism, serotonin transporter, longitudinal study

## Abstract

The genetic predisposition toward suicidal ideation has been explored to identify subgroups at high risk and to prevent suicide. Acute coronary syndrome (ACS) is associated with an increased risk of suicide, but few studies have explored the genetic predisposition toward suicide in ACS populations. Therefore, this longitudinal study explored the genetic predisposition toward suicidal ideation in ACS patients. In total, of 969 patients within 2 weeks after ACS, 711 were followed at 1 year after ACS. Suicidal ideation was evaluated with the relevant items on the Montgomery–Åsberg Depression Rating Scale. Ten genetic polymorphisms associated with serotonergic systems, neurotrophic factors, carbon metabolism, and inflammatory cytokines were examined. Associations between genetic polymorphisms and suicidal ideation within 2 weeks and 1 year of ACS were investigated using logistic regression models. The 5-HTTLPR s allele was significantly associated with suicidal ideation within 2 weeks of ACS after adjusting for covariates and after the Bonferroni correction. TNF-α –308^G/A^, IL-1β –511^C/T^, and IL-1β + 3953C/T were significantly associated with suicidal ideation within 2 weeks after ACS, but these associations did not reach significance after the Bonferroni correction in unadjusted analyses and after adjusting for covariance. However, no significant association between genetic polymorphisms and suicidal ideation was found at 1 year. Genetic predisposition, 5-HTTLPR s allele in particular, may confer susceptibility to suicidal ideation in ACS patients during the acute phase of ACS.

## INTRODUCTION

Suicide, the most devastating outcome of mental health disorders, constitutes a major public health problem. Many studies have explored the etiology of suicide to identify subgroups at high risk. Suicide is suspected to involve numerous biological, psychological, social, and cultural determinants, and the contribution of a genetic predisposition to suicide has been proposed based on the findings of familial heritability and genetic association studies [1].

Physical illnesses are known risk factors, as severe and chronic illnesses are stressful events that may contribute to suicide [2]. Among these, acute coronary syndrome (ACS) is a severe physical illness that is associated with the leading cause of death worldwide; it is also associated with an increased risk of depression, which may increase suicide risk [3]. Furthermore, previous population-based case–control studies found that ACS was strongly associated with an increased risk of suicide per se, and this risk was highest shortly after discharge [4]. Despite the importance of identifying the predictors of suicide in patients with ACS, previous studies on this topic have focused on the psychiatric and clinical predictors of suicidal ideation (SI), including depression, marital status, and higher troponin I levels, as a phenotype [5, 6]. However, few studies have explored the genetic predisposition for suicide in ACS populations.

Suicide is known to be associated with depression and is considered a depressive symptom. However, not all patients with depression suffer from suicidal symptoms and suicidal symptoms represent more severe psychopathology in case of depressed patients with suicidal symptoms, with poorer treatment outcomes and higher subsequent suicidal risk [7]. Therefore, it is worthy investigating genetic predisposition for suicide in patients with ACS. Our previous publications reported that the alleles for the serotonin transporter gene-linked promoter region (5-HTTLPR) s, and brain-derived neurotrophic factor (BDNF) met are associated with depression at the acute phase and its persistence at 1 year whereas proinflammatory cytokine polymorphisms including the tumor necrosis factor-α (TNF-α)-308 A allele and the interleukin (IL)-1β-511 T allele are associated with depression at the acute phase but not at 1 year in ACS [8–11].

Suicide refers to a spectrum of phenomena ranging from suicidal ideation to suicide completion. Suicidal ideation is the first stage before a suicide attempt and is considered the target of preventive efforts given that suicide attempts often occur within a year after the onset of suicidal ideation [12]. Therefore, this longitudinal study of ACS patients study explored the various genetic risk factors for SI, including genetic polymorphisms related to the serotonin system as well as those related to other neurochemical systems.

## MATERIALS AND METHODS

### Study overview

This was performed as a subanalysis using data from a large prospective study of ACS patients, the Korean DEPression in ACS (K-DEPACS) study, which also included a nested randomized controlled trial, the Escitalopram for DEPression in ACS (EsDEPACS) study. A description of the whole K-DEPACS study has been previously published [5], and the organization of these analyses is shown in Supplementary Figure 1. Participants were consecutively recruited from patients recently hospitalized with ACS at the Department of Cardiology of Chonnam National University Hospital, Gwangju, South Korea, which serves as the principal coordinating center for the Korea Acute Myocardial Infarction Registry [13].

In total, 1,152 patients who met the eligibility criteria (see Supplementary Methods) and consented to both participation and blood sampling were included in the acute-phase sample (assessed within 2 weeks after ACS). Participants included in the acute-phase sample were screened as inpatients for depressive disorders by study psychiatrists during the first 2 weeks post-ACS using the Mini-International Neuropsychiatric Interview (MINI) [14]; as outpatients, they were screened every 4 weeks for up to 12 weeks. Of the 378 baseline participants with a diagnosis of depressive disorder, 255 agreed to participate in a 24-week, double-blind, randomized trial of escitalopram or placebo: the EsDEPACS study (ClinicalTrial.gov registry number: NCT00419471). This clinical trial was comprised of six assessments (at baseline and 4, 8, 12, 16, 20, and 24 weeks thereafter), and primary efficacy outcomes were estimated using the Hamilton Depression Rating Scale (HAMD) [15]. The methods and results of this trial, which were reported previously, showed superior therapeutic effects associated with escitalopram, which had no harmful effect compared with placebo [16]. Only conventional medical treatments for ACS were applied to the remaining 123 patients who refused to participate in the trial. All participants assessed within 2 weeks after ACS were approached at 1 year after ACS to evaluate their psychiatric status, including suicidal ideation and depression, during chronic-phase ACS. Written informed consent forms for the K-DEPACS and EsDEPACS studies were gathered from all participants; both studies received ethical approval from the Chonnam National University Hospital Institutional Review Board.

### SI assessment and covariates

SI was assessed within 2 weeks after ACS and at 1 year after ACS using the items on the Montgomery–Åsberg Depression Rating Scale (MADRS-ST) addressing suicidal thoughts [17]. Respondents were asked to rate whether they thought life was worth living or whether they had a suicide plan; scores ranged from 0 (life satisfaction) to 6 (explicit plans for suicide). Following previous studies, the presence of SI was defined by a score of 2 (fleeting suicidal thoughts) or more [18].

Data on the covariates strongly associated with suicide in ACS patients in previous reports [5] were evaluated within 2 weeks after ACS. An assessment was performed to collect information on age, sex, years of education, living status (living alone or not), type of residence (owned or rented), and current occupation (employed or not). To assess patients’ depression characteristics, personal and family histories of depression and DSM-IV depressive disorder (minor + major) using the MINI [14] were evaluated. To assess patients’ cardiovascular risk factors, the following characteristics were also investigated: personal and family histories of ACS, diagnosed hypertension, and diabetes mellitus, hypercholesterolemia according to fasting serum total cholesterol level (> 200 mg/dL) or a history of hyperlipidemia with ongoing treatment, obesity based on measured body mass index (> 25 kg/m^2^), and reported current smoking status. To evaluate comorbid physical conditions, questionnaires asked participants about the presence of chronic kidney disease or chronic obstructive pulmonary disease, as these diseases have been associated with suicide in previous studies [19, 20]. To estimate current cardiac status, ACS severity was evaluated using the Killip classification [21], left ventricular ejection fraction was measured using echocardiography, and heart rate was investigated based on electrocardiography (EKG). Serum cardiac biomarkers, including troponin I and CK-MB, were also assessed. Additionally, major adverse cardiac events (MACE) during the 1-year follow-up were comprised of recurrent myocardial infarction, or recurrent percutaneous coronary intervention and were assessed considering the impact of the post-ACS diagnosis on SI at follow-up.

### Risk-related polymorphisms

10 polymorphisms associated with suicide in the general population [1, 22] were examined: serotonergic systems including serotonin transporter gene-linked promoter region (5-HTTLPR), a variable number of tandem repeats in 5-HTT intron 2 (STin2 VNTR), and serotonin 2a receptor (5-HT2Ra) polymorphisms; brain derived neurotrophic factor (BDNF); proinflammatory cytokine polymorphisms including tumor necrosis factor-α (TNF-α) and interleukin (IL)-1β; and methylenetetrahydrofolate reductase (MTHFR) polymorphisms, which affect homocysteine levels. Polymerase chain reaction (PCR) and PCR-based restriction fragment length polymorphism assays were performed using venous blood. Polymerase chain reaction (PCR) and PCR-based restriction fragment length polymorphism assays were performed. Polymorphism selection and allele detection methods are described in Supplementary Table 1. For the 5-HTTLPR polymorphism, genotypes were categorized as ‘l/l’, ‘l/s’, and ‘s/s’. For the STin2 VNTR polymorphism, the genotypes were categorized as ‘10/12’ and ‘9 or 12/12’ due to the scarcity of the 9 allele frequency (present in only three participants). For the 5-HTR2a 102T/C polymorphism, the genotypes were categorized as ‘*T/T*’, ‘*T/C*’, and ‘*C/C*’. For the 5-HTR2a 1438A/G polymorphism, the genotypes were categorized as ‘G/G’, ‘*G/A*’, and ‘*A/A*’. For BDNF, the genotype was categorized as val/val, val/met, or met/met. For TNF-α -850C/T and -308G/A polymorphisms, ‘*C/C*’, ‘*T/C*’, and ‘*T/T*’ and ‘*G/G*’, ‘*G/A*’, and ‘*A/A*’ were categorized, respectively. For IL-1β, –511C/T genotypes were classified as ‘*C/C*’, ‘*T/C*’, and ‘*T/T*’, whereas +3953C/T genotypes were classified into two groups (*C/C* or *C/T*) considering the infrequency of 3953T/T genotypes. Finally, MTHFR C677T genotypes were classified into ‘*C/C*’, ‘*T/C*’, and ‘*T/T*’.

### Statistical analysis

Demographic and clinical variables were compared between ACS patients with and without SI using *t-*tests or χ^2^ tests, as appropriate. Variables significantly associated SI (*P* < 0.05) were analyzed as covariates in further regression models. Analyses of the association of various genotypes with SI within 2 weeks and at 1 year after ACS were performed using χ^2^ tests comparing allele frequencies associated with the absence or presence of SI. Odds ratios (ORs) for SI related to various genotypes were calculated by applying logistic regression models after adjustment for covariates. Treatment status was considered as a covariate in the follow-up analysis considering previous result of antidepressants on SI [5]. An empirical *p-*value of 0.05 was applied to determine statistical significance. Additionally, Bonferroni’s correction was performed to maintain an overall type 1 error rate of 0.05 in the context of multiple comparisons for 10 polymorphisms: a two-sided *p-*value of 0.005 (0.05/10) was also used to determine statistical significance. To evaluate the effect of individual confounders, additional analyses were conducted using similar regression model with stepwise methods. SPSS for Windows (ver. 21.0; SPSS Inc., Chicago, IL, USA) software was used.

## RESULTS

### Recruitment and rates of SI

The recruitment process and prevalence of suicidal ideation during 1 year are described in Supplementary Figure 1. In total, 969 (84%) members of the K-DEPACS baseline sample (*n* = 1,152) agreed to blood sampling. No significant differences in any covariates, assessed within 2 weeks after ACS, were found between those who agreed to blood collection and those who refused (all *p-*values > 0.15). Of the 969 baseline participants, 711 (73%) ACS patients successfully underwent 1-year follow-up evaluation. Older age and higher Killip class contributed the attrition between 258 patients lost to follow-up and those who followed-up (all *p-*values < 0.05). With respect to the EsDEPACS trial, 255 of the 378 patients (39%) who met criteria for a depressive disorder within 2 weeks after ACS agreed to participate in the EsDEPACS trial (127 randomized to escitalopram and 128 to placebo); those who did not consent (*n* = 123) were treated with standard medical care. Of EsDEPACS participants, 191 (96 escitalopram and 95 placebo) were followed up at 1 year after ACS, whereas 94 participants treated with usual medical care were followed up.

In terms of the prevalence of SI, 195 (20%) of the 960 baseline ACS patients reported SI within 2 weeks after ACS, whereas 87 (12%) of the 711 ACS patients reported SI at 1 year after ACS. SI within 2 weeks after ACS was associated with female, lower educational level, rented housing, current unemployment, personal history of depression, and presence of baseline depression; SI at 1 year after ACS was associated with female, family history of depression, presence of baseline depression, and treatment status (placebo/usual medical care) (Supplementary Table 2).

### Unadjusted genetic associations by suicidal ideation status

Various genotypes were compared according to the SI status (Table 1). The distribution of all genotypes did not deviate from the Hardy–Weinberg equilibrium (all *p-*values > 0.1). SI within 2 weeks after ACS was associated with the 5-HTTLPR s allele, TNF-α -308A allele, and IL-1β -511T and + 3953T alleles, whereas SI at 1 year after ACS was associated with only the 5-HTTLPR s allele. The association of BDNF met allele with SI within 2 weeks after ACS showed only borderline significance (*p* = 0.053). After Bonferroni correction, the only alleles that remained significantly associated with SI within 2 weeks were the 5-HTTLPR s alleles.

**Table 1:** Genotype frequencies by suicidal ideation (SI) status

Polymorphism	Baseline sample			Follow-up sample		
	No SI (*N* = 774)	SI (*N* = 195)	*p*-value	No SI (*N* = 624)	SI (*N* = 87)	*p*-value
5-HTTLPR						
* l/l*	56 (7.2)	5 (2.6)	**< 0.001**	44 (7.1)	1 (1.1)	0.022
* l/s*	292 (37.7)	48 (24.6)		225 (36.1)	25 (28.7)	
* s/s*	426 (55.0)	142 (72.8)		355 (56.9)	61 (70.1)	
STin2 VNTR						
* 9 or 12*	655 (84.6)	156 (80.0)	0.118	526 (84.3)	70 (80.5)	0.363
* 10*	119 (15.4)	39 (20.0)		98 (15.7)	17 (19.5)	
5-HTR2a 1438A/G						
* G/G*	215 (27.8)	44 (22.6)	0.106	159 (25.5)	24 (27.6)	0.563
* G/A*	398 (51.4)	98 (50.3)		331 (53.0)	41 (47.1)	
* A/A*	161 (20.8)	53 (27.2)		134 (21.5)	22 (25.3)	
5-HTR2a 102T/C						
* T/T*	169 (21.8)	39 (20.0)	0.589	132 (21.2)	19 (21.8)	0.969
* T/C*	406 (52.5)	99 (50.8)		334 (53.5)	47 (54.0)	
* C/C*	199 (25.7)	57 (29.2)		158 (25.3)	21 (24.1)	
MTHFR						
* C/C*	275 (35.5)	67 (34.4)	0.943	220 (35.3)	26 (29.9)	0.385
* C/T*	367 (47.4)	95 (48.7)		295 (47.3)	48 (55.2)	
* T/T*	132 (17.1)	33 (16.9)		109 (17.5)	13 (14.9)	
TNF-α -850C/T						
* C/C*	580 (74.9)	135 (69.2)	0.241	462 (74.0)	64 (73.6)	0.548
* C/T*	174 (22.5)	55 (28.2)		146 (23.4)	19 (21.8)	
* T/T*	20 (2.6)	5 (2.6)		16 (2.6)	4 (4.6)	
TNF-α -308G/A						
* G/G*	635 (82.0)	146 (74.9)	0.011	508 (81.4)	69 (79.3)	0.844
* G/A*	125 (16.1)	48 (24.6)		107 (17.1)	17 (19.5)	
* A/A*	14 (1.8)	1 (0.5)		9 (1.4)	1(1.1)	
IL-1β -511C/T						
* C/C*	259 (33.5)	51 (26.2)	0.046	177 (28.4)	21 (24.1)	0.705
* C/T*	359 (46.4)	91 (46.7)		295 (47.3)	43 (49.4)	
* T/T*	156 (20.2)	53 (27.2)		152 (24.4)	23 (26.4)	
IL-1β +3953C/T						
* C/C*	696 (89.9)	164 (84.1)	0.022	540 (86.5)	77 (88.5)	0.612
* C/T*	78 (10.1)	31 (15.9)		84 (13.5)	10 (11.5)	
BDNF						
* val/val*	206 (26.6)	36 (18.5)	0.053	155 (24.8)	18 (20.7)	0.639
* val/met*	392 (50.6)	106 (54.4)		331 (53.0)	47 (54.0)	
* met/met*	176 (22.7)	53 (27.2)		138 (22.1)	22 (25.3)	

Data are represented as n (%) unless otherwise indicated.5-HTTLPR, serotonin transporter gene linked promoter region; STin2 VNTR, serotonin transporter intron 2 variable number tandem repeat; 5-HTR2a, serotonin 2a receptor.

*p*-values were determined using χ^2^ tests. Values in bold type show statistical significance after Bonferroni correction.

### Adjusted genetic associations by suicidal ideation status

Independent associations between various genotypes and SI, adjusted for covariates, are described in Table 2. After adjustment, the association of the 5-HTTLPR genotype with SI within 2 weeks after ACS was strengthened with an increasing number of s alleles and remained significant even after the Bonferroni correction. However, the association with SI at 1 year after ACS fell below statistical significance after adjusting for covariates. There was no significant association between any other genotype and SI either within 2 weeks or at 1 year after ACS. Additional analyses were conducted using a similar regression model as the stepwise method (forward and backward) to evaluate the effect of individual confounders. These results are displayed in Supplementary Tables 3 and 4 (backward data not shown). A significant association was observed between the 5-HTTLPR genotype and SI within 2 weeks following ACS even after the Bonferroni correction whereas the 5-HTTLPR genotype and SI had a borderline significant association at 1 year, although SI was largely affected by baseline depression.

**Table 2:** Adjusted associations of genotype with suicidal ideation

Polymorphism		Baseline sample^a^		Follow-up sample^b^	
		OR (95% CI)	*p*-value	OR (95% CI)	*p*-value
5-HTTLPR	*l/l*	Ref.	**< 0.001**	Ref.	0.064
	*l/s*	1.80 (0.64–5.06)		4.06 (0.53–31.04)	
	*s/s*	3.62 (1.32–9.93)		6.22 (0.83–46.32)	
STin2 VNTR	*9 or 12*	Ref.	0.067	Ref.	0.305
	*10*	1.53 (0.97–2.43)		1.36 (0.76–2.44)	
5-HTR2a 1438A/G	*G/G*	Ref.	0.333	Ref.	0.544
	*G/A*	1.11 (0.72–1.71)		0.79 (0.46–1.37)	
	*A/A*	1.44 (0.87–2.39)		1.05 (0.55–1.99)	
5-HTR2a 102T/C	*T/T*	Ref.	0.753	Ref.	0.913
	*T/C*	0.89 (0.56–1.41)		0.90 (0.50–1.61)	
	*C/C*	1.03 (0.62–1.71)		0.87 (0.44–1.71)	
MTHFR	*C/C*	Ref.	0.977	Ref.	0.376
	*C/T*	1.04 (0.70–1.54)		1.34 (0.80–2.26)	
	*T/T*	1.00 (0.59–1.68)		0.92 (0.45–1.89)	
TNF-α -850C/T	*C/C*	Ref.	0.168	Ref.	0.814
	*C/T*	1.40 (0.93–2.09)		0.98 (0.56–1.71)	
	*T/T*	0.64 (0.21–1.94)		1.45 (0.45–4.75)	
TNF-α -308G/A	*G/G*	Ref.	0.168	Ref.	0.983
	*G/A*	1.27 (0.82–1.95)		0.98 (0.55–1.77)	
	*A/A*	0.20 (0.03–1.65)		0.82 (0.10–6.80)	
IL-1β -511C/T	*C/C*	Ref.	0.507	Ref.	0.849
	*C/T*	1.29 (0.84–1.96)		1.15 (0.66–2.03)	
	*T/T*	1.15 (0.71–1.87)		1.02 (0.53–1.95)	
IL-1β +3953C/T	*C/C*	Ref.	0.065	Ref.	0.611
	*C/T*	1.63 (0.97–2.73)		0.83 (0.41–1.69)	
BDNF	*val/val*	Ref.	0.353	Ref.	0.730
	*val/met*	1.31 (0.83–2.08)		1.09 (0.61–1.97)	
	*met/met*	1.45 (0.86–2.44)		1.30 (0.66–2.55)	

OR, odds ratio; CI, confidence interval; 5-HTTLPR, serotonin transporter gene linked promoter region; STin2 VNTR, serotonin transporter intron 2 variable number tandem repeat; 5-HTR2a, serotonin 2a receptor.

*p*-values for logistic regression likelihood ratio test (df = 1).

^a^Adjusted for sex, education, housing, currently unemployed, personal history of depression, and DSM-IV depression.

^b^Adjusted for sex, family history of depression, DSM-IV depression and intervention.

Values in bold type show statistical significance after Bonferroni correction.

## DISCUSSION

This is the first study to explore genetic associations between ACS and SI using the 1-year longitudinal ACS cohort. Our results confirmed that genetic predisposition played a significant role in SI in ACS patients during the acute phase, whereas the significance of the genetic association was lost during the chronic phase. Among the various genotypes, 5-HTTLPR was significantly associated with SI in ACS during the acute phase, including after adjusting for covariates as well as after Bonferroni correction. Meanwhile, other genotypes including TNF-α -308G/A, IL-1β –511C/T, and IL-1β +3953C/T were significantly associated with SI during the acute phase, but these associations did not reach significance after the Bonferroni correction was applied to unadjusted analyses and after adjusting for covariates.

Our data suggested that genetic vulnerability plays a different role in SI in ACS patients according to time elapsed after ACS. This finding can be explained by the complex etiologies of suicide. Previous reports have suggested that interactions between genetic and environmental factors are important risk factors for suicide [23]. Therefore, it is possible that the genetic effect on SI is more significant during the acute phase (when acute ACS functions as a stressful life event) than during the chronic phase of ACS (when various environmental factors, such as general health and functioning, become stronger determinants of SI). ACS patients often consider ACS to be a serious life event with considerable threat potential immediately after its onset; however, patients have to cope with symptoms, treatments, and lifestyle changes during the chronic phase [24]. During the adaptive phase of ACS, patients are faced with various environmental stresses, such as housework, employment, and social activities, which affect psychological status, including SI and mood [25].

SI within 2 weeks after ACS was associated with the 5-HTTLPR s allele, even after adjustment for covariates, such as depression, and after multiple comparisons. To the best of our knowledge, this is the first genetic association study investigating SI in ACS patients. The 5-HTTLPR has been examined as an important candidate gene for suicidal vulnerability in the general population [1] as it regulates serotonin turnover and its concentrations in the synaptic cleft [26]. Furthermore, it has been suggested that the s allele of 5-HTTLPR is associated with SI in other physical illnesses, stroke [27]. Similarly with previous findings, our results suggested that SI in ACS was also associated with the 5-HTTLPR s allele, especially during the acute phase of ACS. There are several possible mechanisms explaining this association. First, the s allele of 5-HTTLPR is known to be associated with reduced transcriptional activity [26]. The resulting altered serotonergic signaling may, via platelet aggregation, influence the predisposition toward ACS and may mediate cognitive inflexibility and aggressive behavior [28], which would, in turn, impair the ability to cope with stressful events (e.g., an ACS-related event). However, further studies on the underlying mechanisms of dysregulated serotonergic systems related to 5-HTTLPR and SI in ACS are required as other polymorphisms associated with serotonergic function (STin2 VNTR and 5-HTR2a polymorphisms) did not show any significant associations. Second, the 5-HTTLPR s allele is known to be a genetic risk factor for depression in both the general population and ACS patients [29, 30]. Depression is strongly associated with SI, and SI is included in the diagnostic criteria for depression. Therefore, the significant association between 5-HTTLPR and SI in ACS may be associated with the relationship between depression and SI, although further statistical adjustment for depression has been performed. Third, the prevalence of the s allele in our study (76%) was higher than that in Caucasian populations (43%) [29], but it was similar to that reported in East Asian countries (79%) [30]. This ethnic difference may have increased the statistical power to detect associations in our ACS patients.

Additionally, other genotypes associated with cytokine production, including TNF-α -308A, IL-1β –511T, and IL-1β + 3953T, were significantly associated with SI during the acute phase, although the statistical significance of these associations was reduced following Bonferroni correction and following adjustment for covariates. Despite the growing body of evidence showing the role of inflammation in the pathophysiology of suicide [1, 2] the weak positive associations, which were lost after adjustment, may be explained primarily by other factors, such as depression after ACS, as the association between the TNF-α and IL-1β polymorphisms and depression within 2 weeks after ACS was reported previously in our sample [31] as well as in general population [32]. Thus, further studies in ACS patients are required.

The BDNF met allele was not associated with SI during either the acute or the chronic phase of ACS. The BDNF met allele is known to be associated with suicidal behavior in individuals with general depression and with SI in breast cancer patients [1, 33], but no such associations were found with SI in our ACS cohort. These findings may be due to differences in the definition of suicide or in the characteristics of study populations, such as comorbid physical disorders. Further studies using various definitions of suicide and investigations of suicidal ideation in ACS may be required.

Several methodological issues should be considered. First, SI (but not suicide attempts or completed suicides) was investigated as the primary outcome. Although suicidal ideation is considered to correlate with severe suicidal behavior [34], it is difficult to generalize our findings to overall suicidal behavior in ACS patients. However, previous clinical studies in ACS patients and other physically ill patients have investigated suicidal ideation as a phenotype because suicidal ideation is also considered as heritable [2], and more severe types of suicidal behavior are rare in this type of study [6]. Second, suicidal ideation was not defined using a formal, separate instrument, but by the suicide-related items on the MADRS. However, the validity of using suicide-related items from this depression rating scale has proven to be adequate [35], and the approach used in this study has been applied previously [18]. Lastly, attrition in the recruitment process should be considered. Blood sampling for genotyping was available for only 84% of the total ACS patients, although no difference was observed between those who agreed to genotyping and those who did not. Follow up was performed in 73% of baseline participants. Attrition within this group was associated with older age and worse cardiac functioning, and this may have affected the results of the follow-up analyses.

Our study has several strengths. It is the first prospective investigation that additionally benefitted from a nested randomized placebo-controlled trial designed to evaluate associations involving genetic factors, suicidal ideation, and the effect of depression treatment. Various genetic polymorphisms were examined according to suicidal ideation. Moreover, suicidal ideation and other covariates were evaluated at similar time points (within 2 weeks and 1 year after ACS) in a large number of ACS patients. Psychiatric and cardiovascular covariates were assessed using a well-validated assessment scale. The final strength was that participants were recruited consecutively from all eligible patients with recent ACS who were seen in the study hospital, which decreased the possibility of selection bias and increased the generalizability of the findings in terms of the evaluation and treatment of ACS patients.

In conclusion, ACS patients with the 5-HTTLPR s allele were more likely to report SI within 2 weeks but not at 1 year after ACS. SI within 2 weeks of ACS is known to predict poor depressive outcomes [36]; in turn, depression is associated with poor cardiac outcomes. Considering these interactions, identifying ACS patients with greater susceptibility to poor outcomes is important for appropriate management. Additionally, further comprehensive genetic studies are required to detect subgroups of those with ACS who are at high risk for SI, and our findings may provide a basis for the investigation of the genetic risk of suicide in ACS patients.

## SUPPLEMENTARY MATERIALS FIGURE AND TABLES


