# Supplementary material for: Genetic predisposition toward suicidal ideation in patients with acute coronary syndrome
Source: Oncotarget. 2017 Oct 7;8(55):94951–8. doi: 10.18632/oncotarget.21661 (PMC5706927; doi:10.18632/oncotarget.21661)
Supplement: Supplementary file 1 [file oncotarget-08-94951-s001.pdf]

Genetic predisposition toward suicidal ideation in patients with acute coronary syndrome

SUPPLEMENTARY MATERIALS

Eligibility criteria

ACS patients who satisfied the following criteria were eligible for participation in the K-DEPACS study: i) 18–85 years of age; ii) confirmed ACS (the presence of ST-segment elevation myocardial infarction (MI) was determined in patients with > 30 min of continuous chest pain, a new ST-segment elevation ≥ 2 mm on at least two contiguous electrocardiographic leads, and creatine kinase-MB (CK-MB) more than three times normal; the presence of non-ST-segment elevation MI was diagnosed by chest pain and a positive cardiac biochemical marker without new ST-segment elevation; and the presence of unstable angina was determined by chest pain within the preceding 72 h with or without ST-T wave changes or positive cardiac biochemical markers); iii) ability to complete the study questionnaires; iv) ability to understand the study objectives and provide written, informed consent. Exclusion criteria for the K-DEPACS study were: i) occurrence of ACS while hospitalized for another reason; ii) ACS developing < 3 months after a coronary artery bypass graft procedure; iii) uncontrolled hypertension (systolic blood pressure (BP) > 180 mmHg or diastolic BP > 100 mmHg); iv) resting heart rate < 40/min; v) severe physical illnesses threatening life or interfering with the recovery from ACS; vi) persistent clinically significant laboratory abnormalities.

Additional inclusion criteria for the EsDEPACS trial were: i) Beck Depression Inventory (Beck et al., 1961) score > 10 and ii) confirmation of a major or minor depressive disorder according to the DSM-IV criteria. Additional exclusion criteria for the EsDEPACS trial were: i) concomitant use of class I antiarrhythmic medications, reserpine, guanethidine, clonidine, methyldopa, lithium, anticonvulsants, antipsychotics, or antidepressants; ii) a history of neuropsychiatric illnesses such as dementia, Parkinson’s disease, brain tumor, psychosis, bipolar disorder, alcoholism, or other substance dependence; iii) pregnancy; and iv) participation in other drug trials.

REFERENCES

1. Beck AT, Ward CH, Mendelson M, Mock J, Erbaugh J. An inventory for measuring depression. Arch Gen Psychiatry. 1961; 4:561–571.

Supplementary Table 1: Polymerase chain reaction (PCR) methods for allele detection

Polymorphism	Forward (F) and reverse (R) primer	Restriction enzyme	Allele	Allele Frequency		
				Present study	East Asian	Caucasian
5-HTTLPR	F: 5′-GGCGTTGCCGCTCTGAATGC-3’	–	l	0.24	0.21	0.57
	R: 5′-GAGGGACTGAGCTGGACAACCAC-3’	–	s	0.76	0.79 [1]	0.43 [2]
STin2 VNTR	F: 5′-GTCAGTATCACAGGCTGCGAG-3’	–	9or12	0.84	0.91	0.61
	R: 5′-TGTTCCTAGTCTTACGCCAGTGG-3’		10	0.16	0.09 [3]	0.39 [4]
5-HTR2a 1438A/G	F: 5′-ACTGCGAAACCAACTTATTTCC-3’	*MSp*I	1438*A*	0.52	0.50	0.47
	R: 5′-CTTGTGCAGATTCCCATTAAGG-3’		1438*G*	0.48	0.50 [5]	0.52 [6]
5-HTR2a 102T/C	F: 5′-TCTGCTACAAGTTCTGGCTTC-3’	*Hpa*II	102*T*	0.48	0.67	0.37
	R: 5′-CTGCAGCTTTTTCTCTAGGGG-3’		102*C*	0.52	0.33 [7]	0.63 [8]
BDNF	F: 5′-ACTCTGGAGAGCGTGAATGG-3’	*Eco*72I	Val	0.51	0.55	0.79
	R: 5′-ACTACTGAGCATCACCCTGGA-3’		met	0.49	0.45 [9]	0.21 [10]
TNF-α -850C/T	F: 5′-TCGAGTATCGGGGACCCCCCGTT-3’	*Hinc* II	−850*C*	0.86	0.87	0.75
	R: 5′-CCAGTGTGTGGCCATATCTTCTT-3’		–850*T*	0.14	0.13 [11]	0.25 [12]
TNF-α -308G/A	F: 5′-AGGCAATAGGTTTTGAGGGCCAT-3’	*Nco* I	–308*G*	0.90	0.98	0.85
	R: 5′-TCCTCCCTGCTCCGATTCCG-3’		–308*A*	0.10	0.02 [13]	0.15 [14]
IL-1β -511C/T	F: 5′-GCCTGAACCCTGCATACCGT-3’	*Ava* I	–511*C*	0.55	0.55	0.61
	R: 5′-GCCAATAGCCCTCCCTGTCT-3’		–511*T*	0.45	0.45 [15]	0.31 [16]
IL-1β +3953C/T	F: 5′-CTCAGGTGTCCTCGAAGAAATCAAA-3’	*Taq* I	+3935*C*	0.94	0.96	0.75
	R: 5′-GCTTTTTTGCTGTGAGTCCCG-3’		+3935*T*	0.06	0.04 [17]	0.25 [14]
MTHFR	F: 5′-GCACTTGAAGGAGAAGGTGTCTG-3′	*Hin*FI	*677C*	0.59	0.64	0.69
	R: 5′-AGCTGCGTGATGATGAAATCG-3′		*677T*	0.41	0.36 [18]	0.31[19]

5-HTTLPR = serotonin transporter gene linked promoter region; STin2 VNTR = serotonin transporter intron 2 variable number tandem repeat; 5-HTR2a = serotonin 2a receptor.

REFERENCES

1. Nakatani D, Sato H, Sakata Y, Shiotani I, Kinjo K, Mizuno, H, Shimizu M, Ito H, Koretsune Y, Hirayama A, Hori M, Osaka Acute Coronary Insufficiency Study Group. 2005. Influence of serotonin transporter gene polymorphism on depressive symptoms and new cardiac events after acute myocardial infarction. Am Heart J. 2005; 150:652–658.
2. Otte C, McCaffery J, Ali S, Whooley MA. Association of a serotonin transporter polymorphism (5-HTTLPR) with depres- sion, perceived stress, and norepinephrine in patients with coronary disease: the Heart and Soul Study. Am J Psychiatry. 2007; 164:1379–1384.
3. Kunugi H, Hattori M, Kato T, Tatsumi M, Sakai T, Sasaki T, Hirose T, Nanko S. Serotonin transporter gene polymorphisms: ethnic difference and possible association with bipolar affective disorder. Mol Psychiatry. 1997; 2:457–462.
4. Mellerup E, Bennike B, Bolwig T, Dam H, Hasholt L, Jorgensen MB, Plenge P, Sørensen SA. Platelet serotonin transporters and the transporter gene in control subjects, unipolar patients and bipolar patients. Acta Psychiatr Scand . 2001; 103:229–233.
5. Ono H, Shirakawa O, Nishiguchi N, Nishimura A, Nushida H, Ueno Y, Maeda K. Serotonin 2A receptor gene polymorphism is not associated with completed suicide. J Psychiatry Res. 2001; 35:173–176.
6. Saiz PA, García-Portilla P, Paredes B, Corcoran P, Arango C, Morales B, Sotomayor E, Alvarez V, Coto E, Flórez G, Bascaran MT, Bousoño M, Bobes J. Role of serotonergic-related systems in suicidal behavior: data from a case-control association study. Prog Neuropsychopharmacol Biol Psychiatry. 2011; 35:1518–1524.
7. Chen RY, Sham P, Chen EY, Li T, Cheung EF, Hui TC, Kwok CL, Lieh-Mak F, Zhao JH, Collier D, Murray R. No Association between T102C polymorphism of serotonin-2A receptor gene and clinical phenotypes of Chinese Schizophrenic patients. Psychiatry Res. 2001; 105:175–185.
8. Wrzosek M, Łukaszkiewicz J, Wrzosek M, Serafin P, Jakubczyk A, Klimkiewicz A, Matsumoto H, Brower KJ, Wojnar M. Association of polymorphisms in HTR2A, HTR1A and TPH2 genes with suicide attempts in alcohol dependence: a preliminary report. Psychiatry Res. 2011; 190:149–151.
9. Huang TL, Lee CT. Associations between brain-derived neurotrophic factor G196A gene polymorphism and clinical phenotypes in schizophrenia patients. Chang Gung Med J. 2007; 30:408–413.
10. Pregelj P, Nedic G, Paska AV, Zupanc T, Nikolac M, Balažic J, Tomori M, Komel R, Seler DM, Pivac N. The association between brain-derived neurotrophic factor polymorphism (BDNF Val66Met) and suicide. J Affect Disord. 2011; 128:287–290.

11. Zhao Y, Xia S, Zou L. The Association between Polymorphism of TNF-α Gene and Hypertensive Disorder Complicating Pregnancy. J Huazhong Univ Sci and Technolog Med Sci. 2007; 27:729–732.

12. McCusker SM, Curran MD, Dynan KB, McCyllagh CD, Urquhart DD, Middleton D, Patterson CC, McIlroy SP, Passmore AP. Association between polymorphism in regulatory region of gene encoding tumour necrosis factor alpha and risk of Alzheimer's disease and vascular dementia: a case-control study. Lancet. 2001; 357:436–439.

13. Higuchi T, Seki N, Kamizono S, Yamada A, Kimura A, Kato H, Itoh K. Polymorphism of the 5′-flanking region of the human tumor necrosis factor (TNF)-a gene in Japanese. Tissue Antigens. 1998; 51:605–612.

14. Misener VL, Gomez L, Wigg KG, Luca P, King N, Kiss E, Daróczi G, Kapornai K, Tamas Z, Mayer L, Gádoros J, Baji I, Kennedy JL, Kovacs M, Vetró A, Barr CL; International Consortium for Childhood-Onset Mood Disorders. Cytokine genes TNF , IL1A , IL1B, IL6 , IL1RN and IL10, and childhood-onset mood disorders. Neuropsychobiol. 2008; 58:71–80.

15. Yu YW, Chen TJ, Hong CJ, Chen HM, Tsai SJ. Association study of the interleukin-1 beta (C-511T) genetic polymorphism with major depressive disorder, associated symptomatology, and antidepressant response. Neuropsychopharmacology. 2003; 28;1182–1185.

16. Tadic A, Rujescu D, Muller MJ, Kohnen R, Stassen HH, Szegedi A, Dahmen N. Association analysis between variants of the interleukin-1beta and the interleukin-1 receptor antagonist gene and antidepressant treatment response in major depression. Neuropsychiatr Dis Treat. 2008; 4:269–276.

17. Mu Y, Liu J, Wang B, Wen Q, wang J, Yan J, et al. Interleukin 1 beta (IL-1b) promoter C [-511] T polymorphism but not C [+3953] T polymorphism is associated with polycystic ovary syndrome. Endocrine. 2010; 37:71–75.

18. Shen X, Wu Y, Guan T, Wang X, Qian M, Lin M, Shen Z, Sun J, Zhong H, Yang J, Li L, Yuan Y). Association analysis of COMT/MTHFR polymorphisms and major depressive disorder in Chinese Han population. J Affect Disord. 2014; 161:73–78.

19. Chojnicka I, Sobczyk-Kopcioł A, Fudalej M, Fudalej S, Wojnar M, Waśkiewicz A, Broda G, Strawa K, Pawlak A, Krajewski P, Płoski R. No association between MTHFR C677T polymorphism and completed suicide. Gene. 2012; 511:118–121.

Supplementary Table 2: Baseline sociodemographic and clinical characteristics by suicidal ideation (SI) status

	Baseline sample (N = 969)			Follow-up sample (N = 711)		
	No SI (N = 774)	SI (N = 195)	*p*-value	No SI (N = 624)	SI (N = 87)	*p*-value
**Sociodemographic characteristics**						
Age, mean (SD) years	58.0 (11.3)	58.9 (10.6)	0.315	57.6 (10.7)	57.4 (11.1)	0.855
Sex, *N* (%) female	201 (26.0)	68 (34.9)	**0.013**	163 (26.1)	33 (37.9)	**0.021**
Education, mean (SD) year	10.0 (4.7)	9.1 (4.4)	**0.012**	10.0 (4.6)	9.2 (4.8)	0.118
Living alone, *N* (%) yes	71 (9.2)	21 (10.8)	0.497	49 (7.9)	11 (12.6)	0.132
Housing, *N* (%) rented	109 (14.1)	41 (21.0)	**0.017**	102 (16.3)	20 (23.0)	0.124
Currently unemployed, *N* (%)	279 (36.0)	89 (45.6)	**0.014**	213 (34.1)	38 (43.7)	0.081
**Depression characteristics**						
Personal history of depression, *N* (%)	19 (2.5)	15 (7.7)	**< 0.001**	24 (3.8)	6 (6.9)	0.247
Family history of depression, *N* (%)	15 (1.9)	8 (4.1)	0.108	14 (2.2)	6 (6.9)	**0.026**
DSM-IV depression, *N* (%)	221 (28.6)	157 (80.5)	**< 0.001**	232 (37.2)	53 (60.9)	**< 0.001**
**Cardiac risk factors, *N* (%)**						
Personal history of ACS	30 (3.9)	9 (4.6)	0.639	25 (4.0)	6 (6.9)	0.255
Family history of ACS	24 (3.1)	7 (3.6)	0.729	18 (2.9)	6 (6.9)	0.103
Hypertension	360 (46.5)	98 (50.3)	0.349	282 (45.2)	42 (48.3)	0.588
Diabetes mellitus	144 (18.6)	47 (24.1)	0.085	118 (18.9)	24 (27.6)	0.058
Hypercholesterolemia	384 (49.6)	102 (52.3)	0.501	333 (53.4)	50 (57.5)	0.472
Obesity	341 (44.1)	74 (37.9)	0.123	280 (44.9)	35 (40.2)	0.414
Current smoker	297 (38.4)	69 (35.4)	0.442	247 (39.6)	33 (37.9)	0.768
**Comorbidities**						
Chronic kidney disease	13 (1.4)	0 (0)	0.085	8 (1.1)	0 (0)	0.605
Chronic obstructive pulmonary disease	9 (1.0)	4 (1.8)	0.301	10 (1.4)	0 (0)	0.620
**Current cardiac status**						
Killip class >1, *N* (%)	132 (17.1)	36 (18.5)	0.643	101 (16.2)	13 (14.9)	0.767
LVEF, mean (SD) %	61.2 (11.4)	61.1 (10.8)	0.911	61.4 (11.0)	59.7 (11.8)	0.183
Heart rate, mean (SD) beat/min	74.7 (12.1)	76.1 (15.8)	0.230	75.1 (12.7)	74.5 (13.7)	0.710
Troponin I, mean (SD) mg/dL	9.5 (15.0)	11.5 (14.7)	0.092	10.2 (15.7)	10.9 (17.4)	0.724
CK-MB, mean (SD) mg/dL	16.8 (38.2)	19.7 (33.3)	0.333	18.1 (39.1)	16.6 (35.5)	0.736
**Intervention group, *N* (%)**						
Escitalopram				82 (35.3)	14 (26.4)	**0.001**
Placebo				74 (31.9)	21 (39.6)	
Non-participants				76 (32.8)	18 (34.0)	
**Post-ACS diagnosis**						
Recurrent MI or PCI				62 (8.5)	8 (11.4)	0.837

[a]*p*-values were determined using *t*-tests or χ^2 tests as appropriate.
ACS = acute coronary syndrome, LVEF = left ventricular ejection fraction, CK-MB = creatine kinase-MB, MI = myocardial infarction, PCI = percutaneous coronary intervention.
Values in bold type show statistical significance ($P < 0.05$).

Supplementary Table 3: Adjusted associations of genotype with suicidal ideation at baseline: forward stepwise methods

Polymorphism		Baseline sample[a]		
		Walds	OR (95% CI)	*p*-value
Step 1:				
DSM-IV depression		139.84	10.34 (7.02–15.23)	< 0.001
Step 2:				
5-HTTLPR	*l/l*	Ref.		< 0.001
	l/s	1.04	1.70 (0.61–4.70)	0.310
	s/s	5.81	3.37 (1.25–9.03)	0.016
DSM-IV depression		135.97	10.19 (6.90–15.06)	< 0.001
Step3:				
Past depression		9.36	3.66 (1.59–8.39)	0.002
5-HTTLPR	*l/l*	Ref.		< 0.001
	l/s	1.08	1.73 (0.62–4.84)	0.298
	s/s	5.96	3.47 (1.28–9.41)	0.015
DSM-IV depression		134.47	10.23 (6.91–15.16)	< 0.001

OR, odds ratio; CI, confidence interval; 5-HTTLPR, serotonin transporter gene linked promoter region; STin2 VNTR, serotonin transporter intron 2 variable number tandem repeat;
p-values for logistic regression likelihood ratio test (df = 1).
[a]Adjusted for sex, education, housing, currently unemployed, personal history of depression, and DSM-IV depression.

Supplementary Table 4: Adjusted associations of genotype with suicidal ideation at the follow-up: forward stepwise methods

Polymorphism		Follow-up sample[b]		
		**Walds**	**OR (95% CI)**	***p*-value**
**Step1:**				
DSM-IV depression		17.01	2.63 (1.66–4.17)	< 0.001
**Step 2:**				
DSM-IV depression		15.93	2.56 (1.62–4.07)	< 0.001
Family depression		3.93	2.77 (1.01–7.55)	0.047
**Step 3:**				
DSM-IV depression		14.37	2.46 (1.54–3.91)	< 0.001
5-HTTLPR	l/l	Ref.		0.065
	l/s	1.84	4.09 (0.54–31.20)	0.175
	s/s	3.71	6.23 (0.84–46.36)	0.074
Family depression		16.07	2.69 (0.98–7.37)	0.054

OR, odds ratio; CI, confidence interval; 5-HTTLPR, serotonin transporter gene linked promoter region; STin2 VNTR, serotonin transporter intron 2 variable number tandem repeat;
p-values for logistic regression likelihood ratio test (df = 1).
[b]Adjusted for sex, family history of depression, DSM-IV depression, and intervention.

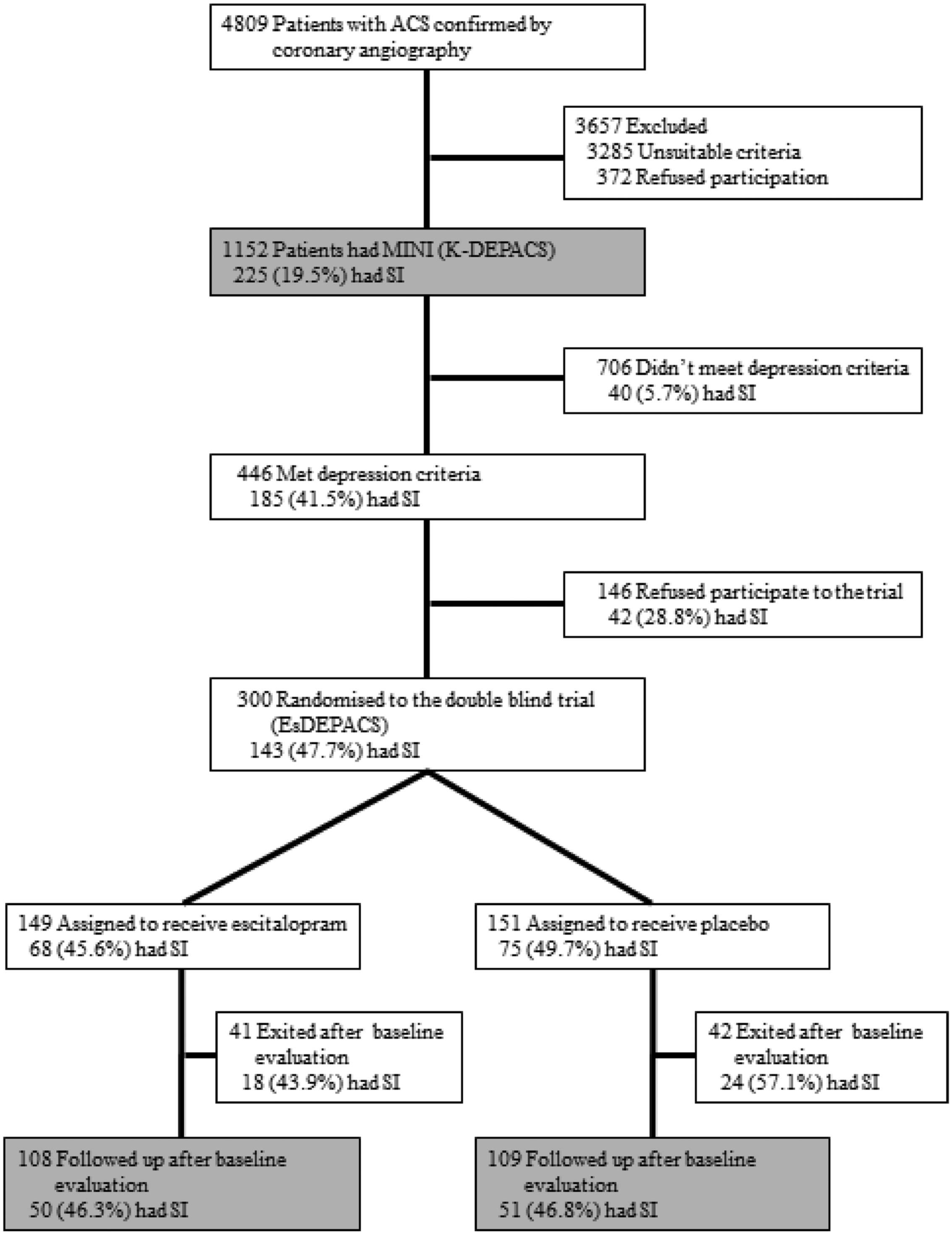

Supplementary Figure 1: The recruitment process and prevalence of suicidal ideation during 1 year. ACS = acute coronary syndrome; MINI = Mini-International Neuropsychiatric Interview; SI = Suicidal ideation; K-DEPACS = Korean DEPression in Acute Coronary Syndrome study; EsDEPACS = Escitalopram for DEPression in Acute Coronary Syndrome study.
